# Serum metabolic signatures of coronary and carotid atherosclerosis and subsequent cardiovascular disease

**DOI:** 10.1093/eurheartj/ehz235

**Published:** 2019-05-18

**Authors:** Ioanna Tzoulaki, Raphaële Castagné, Claire L Boulangé, Ibrahim Karaman, Elena Chekmeneva, Evangelos Evangelou, Timothy M D Ebbels, Manuja R Kaluarachchi, Marc Chadeau-Hyam, David Mosen, Abbas Dehghan, Alireza Moayyeri, Diana L Santos Ferreira, Xiuqing Guo, Jerome I Rotter, Kent D Taylor, Maryam Kavousi, Paul S de Vries, Benjamin Lehne, Marie Loh, Albert Hofman, Jeremy K Nicholson, John Chambers, Christian Gieger, Elaine Holmes, Russell Tracy, Jaspal Kooner, Philip Greenland, Oscar H Franco, David Herrington, John C Lindon, Paul Elliott

**Affiliations:** 1 Department of Epidemiology and Biostatistics, School of Public Health, Imperial College London, Norfolk Place, London, UK; 2 MRC-PHE Centre for Environment and Health, School of Public Health, Imperial College London, Norfolk Place, London, UK; 3 Department of Hygiene and Epidemiology, University of Ioannina Medical School, University Campus Road 455 00, Ioannina, Greece; 4 Dementia Research Institute, Imperial College London, Norfolk Place, London, UK; 5 LEASP, UMR 1027, Inserm-Université Toulousse III Paul Sabatier, Toulousse, France; 6 Metabometrix Ltd, Imperial Incubator, Bessemer Building, Prince Consort Road, London, UK; 7 Division of Computational and Systems Medicine, Department of Surgery and Cancer, Imperial College London, South Kensington Campus, London, UK; 8 Farr Institute of Health Informatics Research, University College London Institute of Health Informatics, 222 Euston Road, London, UK; 9 MRC Integrative Epidemiology Unit, School of Social and Community Medicine, University of Bristol, Oakfield House, Oakfiled Grove, Bristol, UK; 10 Department of Pediatrics, Institute for Translational Genomics and Population Sciences, Los Angeles Biomedical Research Institute at Harbor-UCLA Medical Center, 1000 W Carson St, Torrance, CA, USA; 11 Department of Medicine, Institute for Translational Genomics and Population Sciences, Los Angeles Biomedical Research Institute at Harbor-UCLA Medical Center, 1000 W Carson St, Torrance, CA, USA; 12 Department of Epidemiology, Erasmus University Medical Center, University Medical Center Rotterdam, CA Rotterdam, the Netherlands; 13 Department of Epidemiology, Human Genetics, and Environmental Sciences, Human Genetics Center, School of Public Health, The University of Texas Health Science Center at Houston, 1200 Pressler Street, Houston, TX, USA; 14 Department of Epidemiology, Harvard T.H. Chan School of Public Health, 677 Huntington Avenue, Boston, MA, USA; 15 London North West Healthcare NHS Trust, Northwick Park Hospital, Watford Rd, Harrow, UK; 16 German Research Centre for Environmental Health, Helmholtz Zentrum München, Ingolstädter Landstraße 1, D Neuherberg, Germany; 17 M.D. College of Medicine University of Vermont, The Robert Larner, Given Medical Bldg, E-126, 89 Beaumont Ave, Burlington, VT, USA; 18 National Heart & Lung Institute, Faculty of Medicine, Imperial College London, Guy Scadding Building, Dovehouse St, Chelsea, London, UK; 19 Department of Preventive Medicine, Northwestern University, Feinberg School of Medicine, 680 North Lake Shore Drive, Suite, 1400, Chicago, IL, USA; 20 Institute of Social and Preventive Medicine (ISPM), University of Bern, Mittelstrasse 43, Bern, Switzerland; 21 Section on Cardiovascular Medicine, Department of Internal Medicine, Wake Forest University School of Medicine, Medical Center Boulevard, Winston-Salem, NC, USA

**Keywords:** Atherosclerosis, Metabolomics, Metabolic phenotyping, Coronary artery calcium, Intima-media thickness, Epidemiological studies

## Abstract

**Aims:**

To characterize serum metabolic signatures associated with atherosclerosis in the coronary or carotid arteries and subsequently their association with incident cardiovascular disease (CVD).

**Methods and results:**

We used untargeted one-dimensional (1D) serum metabolic profiling by proton nuclear magnetic resonance spectroscopy (^1^H NMR) among 3867 participants from the Multi-Ethnic Study of Atherosclerosis (MESA), with replication among 3569 participants from the Rotterdam and LOLIPOP studies. Atherosclerosis was assessed by coronary artery calcium (CAC) and carotid intima-media thickness (IMT). We used multivariable linear regression to evaluate associations between NMR features and atherosclerosis accounting for multiplicity of comparisons. We then examined associations between metabolites associated with atherosclerosis and incident CVD available in MESA and Rotterdam and explored molecular networks through bioinformatics analyses. Overall, 30 ^1^H NMR measured metabolites were associated with CAC and/or IMT, *P *=* *1.3 × 10^−14^ to 1.0 × 10^−6^ (discovery) and *P *=* *5.6 × 10^−10^ to 1.1 × 10^−2^ (replication). These associations were substantially attenuated after adjustment for conventional cardiovascular risk factors. Metabolites associated with atherosclerosis revealed disturbances in lipid and carbohydrate metabolism, branched chain, and aromatic amino acid metabolism, as well as oxidative stress and inflammatory pathways. Analyses of incident CVD events showed inverse associations with creatine, creatinine, and phenylalanine, and direct associations with mannose, acetaminophen-glucuronide, and lactate as well as apolipoprotein B (*P *<* *0.05).

**Conclusion:**

Metabolites associated with atherosclerosis were largely consistent between the two vascular beds (coronary and carotid arteries) and predominantly tag pathways that overlap with the known cardiovascular risk factors. We present an integrated systems network that highlights a series of inter-connected pathways underlying atherosclerosis.

**Figure:**
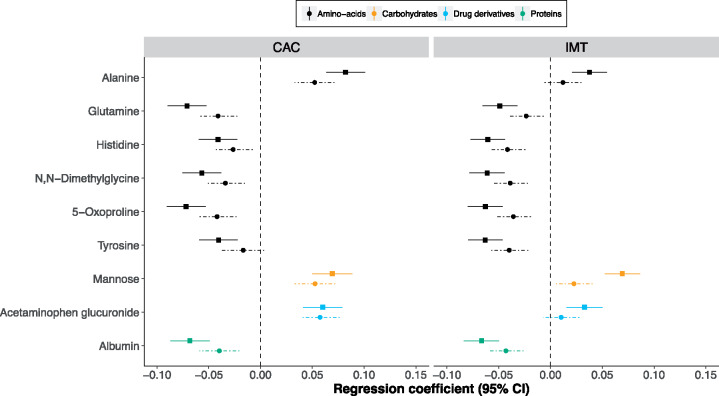

## Introduction

Atherosclerosis and its clinical complications, mainly cardiovascular disease (CVD), is a leading cause of death and disability worldwide.^1^^,^^2^ Epidemiological and laboratory research have provided insight into the molecular mechanisms associated with the pathophysiology of atherosclerosis.^3–5^ Conventional cardiovascular risk factors (such as smoking, hypertension, and dyslipidaemia) are well-established. However, it is not well understood how these risk factors mechanistically cause atherosclerosis and whether independent pathways exist that bypass these conventional factors altogether.^6^ Such further understanding of the molecular pathways underlying atherosclerotic disease may facilitate novel strategies which interrupt, reverse, or prevent its initiation prior to clinical disease. Untargeted metabolic phenotyping (metabolomics), through the simultaneous measurement of a wide range of low molecular weight molecules in biological samples, offers the opportunity to provide a high-resolution picture of biological signatures underlying complex traits as well as potential for new prognostic and diagnostic markers.^7^ In this study, we examined the serum metabolic signature of measures of subclinical atherosclerosis in carotid and coronary arteries using untargeted metabolic profiling by high-resolution proton nuclear magnetic resonance (^1^H NMR) spectroscopy in the Multi-Ethnic Study of Atherosclerosis (MESA) cohort, with replication in independent data from the Rotterdam and the London Life Sciences Prospective Population Study (LOLIPOP) studies. We also performed some additional analyses. First, we examined whether replicated metabolites were independent of main cardiovascular risk factors. We also tested these metabolites against incident CVD events regardless of their independence with cardiovascular risk factors. Finally, we performed gene and molecular network mapping to investigate the molecular pathways underlying their association with subclinical and clinically manifest atherosclerosis.

## Methods

### Study samples

Metabolic profiling was performed on stored serum samples randomly selected from the baseline clinic exams of three population-based cohorts: MESA (*N* = 3867), LOLIPOP (*N* = 1917), and Rotterdam Study (*N* = 1652). In brief, MESA is a prospective cohort of 6814 US participants aged 45 to 84 years recruited via six field centres between 2000 and 2002.^8^ Participants were free of known CVD at baseline and were recruited from four ethnicities (White, African-American, Chinese-American, and Hispanic). The Rotterdam Study is a prospective cohort study in the Ommoord district of the city of Rotterdam, the Netherlands. At baseline, between 1990 and 1993, 7983 participants over 55 years old were interviewed at home and underwent extensive clinical examination at the research centre.^9^ The third visit took place between 1997 and 1999, and included 4797 participants. LOLIPOP is a prospective cohort study of 28 372 people from 35 to 74 years old living in West London, UK from two main ethnic groups (European and South Asian) recruited between 2002 and 2008.^10^

Subclinical atherosclerosis was assessed by coronary artery calcium (CAC) measured by computerized tomography, quantified using the Agatston score, and intima-media thickness (IMT)^11–13^ measured by carotid ultrasound at baseline clinic exams in all three cohorts. In each cohort, we used the mean of the maximum far wall IMT from scans of the right and left common carotid arteries using comparable protocols. Standard cardiovascular risk factors and biomarkers, along with data on treatment and lifestyle characteristics were obtained in all three cohorts as previously described.^5–7^ Cardiovascular disease events [myocardial infarction (MI) and stroke] were available in MESA [after median 10 years of follow-up] and the Rotterdam Study [after median 11 years of follow-up].^8^^,^^14^^,^^15^ Study design is summarized in Supplementary material online, *Figure S1*.

### Proton nuclear magnetic resonance metabolic profiling

Nuclear magnetic resonance measurements were carried out using a previously published protocol^16^ (see Supplementary material online, for expanded Methods section). A standard ^1^H NMR one-dimensional (1D NMR) spectrum with water suppression (also called the NOESY-presat sequence) and a T2-edited spectrum using the Carr-Purcell-Meiboom-Gill (CPMG) sequence were obtained for each sample. The standard ^1^H NMR spectrum detects signatures of all proton containing compounds, with the resultant spectrum comprising sharp peaks for small molecule species, broad bands from the lipoproteins and a largely featureless background from proteins. The CPMG experiment exploits the variation in the nuclear spin relaxation times of the large and small molecules to reduce the intensities of the broad signals from the large compounds (proteins and lipoproteins) producing a spectrum with a flatter baseline and mainly small molecule metabolite peaks. The acquisition parameters of each experiment are detailed in the Supplementary material online. The spectral processing was performed using the software TOPSPIN 3.1 (Bruker Biospin, Rheinstetten, Germany). For each spectrum, the free induction decay underwent a zero filling by a factor of two and a line broadening of 0.3 Hz producing 128K frequency domain points prior to Fourier transformation. The spectra were then automatically phased and baseline corrected, and the chemical shifts were calibrated to the glucose signal at 5.233 ppm. Spectral data were imported into MATLAB [Version 8.3 (R2014a) Mathworks Inc., Natwick, MA, USA] for further processing. The ^1^H NMR spectroscopic analysis was completed in six batches corresponding to the three cohorts and two ^1^H NMR measurement (experimental) phases. The processing workflow to integrate the multicohort ^1^H NMR metabolic profiling data has previously been described^17^ and further details are in Supplementary material online, Data Supplement.

### Proton nuclear magnetic resonance lipoprotein profiles

We quantified lipoprotein subclasses from the MESA ^1^H NMR data based on the deconvolution of the methyl (at 0.84–0.93 ppm) and methylene resonances (at 1.22–1.26 ppm) using a proprietary procedure (developed by Bruker Biospin, Rheinstetten, Germany) adapted from Petersen *et al*.^18^ To assess measurement quality, the Pearson correlation coefficients between conventional measurements and the Bruker ^1^H NMR-derived values for total high-density lipoprotein (HDL) and low-density lipoprotein (LDL) and triglycerides were calculated. Analysis of 105 lipoprotein subclasses was carried out including different chemical components of intermediate-density lipoprotein (density 1.006–1.019 kg/L), very low-density lipoproteins (VLDL, 0.950–1.006 kg/L), LDL (density 1.09–1.63 kg/L), and HDL (density 1.063–1.210 kg/L). The LDL sub-fraction was fractionated into six density classes (LDL-1 1.019–1.031 kg/L, LDL-2 1.031–1.034 kg/L, LDL-3 1.034–1.037 kg/L, LDL-4 1.037–1.040 kg/L, LDL-5 1.040–1.044 kg/L, and LDL-6 1.044–1.063 kg/L) and the HDL sub-fraction in four density classes (HDL-1 1.063–1.100 kg/L, HDL-2 1.100–1.125 kg/L, HDL-3 1.125–1.175 kg/L, and HDL-4 1.175–1.210 kg/L).^18^^,^^19^

### Metabolite identification

To help with identification of peaks in the ^1^H NMR data, reduction using a semi-automatic clustering of the full resolution ^1^H NMR spectrum (30 590 features) was performed using the statistical recoupling of variables (SRV).^20^ The algorithm identifies clusters of 10 or more consecutive resonance features with a correlation of *r* ≥0.9; clusters could also be grouped into a supercluster if the correlation with the neighbouring cluster was *r* = 0.9 or above. To optimize the efficiency of SRV, superclusters were generated from the aggregation of a maximum of three clusters according to Blaise *et al*.^21^ Each cluster was then manually checked (blind to knowledge of atherosclerosis status) to improve the groupings and identify peak overlaps. Thus, 132 clusters were identified in ^1^H NMR standard 1D and 157 clusters in CPMG data, each of them corresponding to a single peak or a group of peaks.

The chemical shift (in ppm), the coupling constant value (J in Hz), the peak multiplicity (singlet, doublet, and multiplet), and peak connectivity of the NMR signals of interest were identified using 1D and 2D [2D JRES, COrrelation SpectroscopY (COSY), TOtal Correlation SpectroscopY (TOCSY), Heteronuclear single quantum correlation spectroscopy (HSQC)] NMR experiments and statistical correlation methods [STOCSY (Statistical Total Correlation Spectroscopy) and STORM (Subset Optimisation by Reference Matching)].^22^^,^^23^ This information was then compared with available in-house and publicly available databases (Human Metabolome Database^24^) as well as with published data on human serum and plasma metabolite components. The metabolite identities were confirmed by spike-in experiments when the chemical standards were available (Supplementary material online, *Figures S2–S5*). The level of peak overlap in the clusters of interest and the level of confidence in the assignment of the identified metabolites were adapted from Sumner *et al*.^25^ The metabolite assignment is as follows: (i) compound identified with spiking, (ii) annotated compounds (without chemical reference standards, based upon physicochemical properties, and/or spectral similarity with public/commercial spectral libraries), (iii) putatively characterized compound classes (e.g. based upon characteristic physicochemical properties of a chemical class of compounds, or by spectral similarity to known compounds of a chemical class), and (iv) unknown compounds (a) well-resolved peaks which can be differentiated and quantified based upon spectral data; (b) overlapped or poorly resolved peaks, from which signal differentiation and quantification may be compromised.

### Statistical analysis

#### Main analysis

CAC (Agatston score) was transformed to ln(CAC + 1) and IMT to log_10_(IMT) for all analyses. We first carried out analyses in the MESA study data and then took forward significantly associated features for replication in the Rotterdam Study and LOLIPOP data. In MESA, for each of the 30 590 spectral features, we carried out a linear regression against each of CAC and IMT with adjustment for age, gender, ethnicity, and measurement phase (Model 1). Discovery models used partial Spearman correlations between CAC or IMT and metabolic features along with *P* values to indicate the strength of associations. We also presented results as standardized regression coefficients (per standard deviation) from linear regression. Each of the 30 590 spectral features were standardized (mean-centred and scaled to unit variance) when standardized beta coefficients were presented. To take into account the high degree of correlation in spectral data, we used a permutation-based method to estimate across the three cohort studies the Metabolome Wide Significance Level (MWSL or α').^26^^,^^27^ For each permutation, we randomly allocate the outcome of interest to each study participant across cohort studies to mimic the null hypothesis of no association; we then calculate the *P*-value for each spectral variable using a linear regression model as described above and record the lowest *P*-value. We ran 10 000 permutations per outcome (CAC or IMT) for both 1D NMR and CPMG spectra. The per-variable significance level α' giving a 5% Family-Wise Error Rate (FWER, α) corresponds to the 500th of these lowest *P-*values. The effective number of tests (ENT) is defined as the number of independent tests that would be required to obtain the same significance level using Bonferroni correction: ENT = α/α'. We computed *P*-value thresholds for each combination of model, outcome, and ^1^H NMR assay type, as summarized in Supplementary material online, *Table S1* and defined a unified threshold derived from the median ENT for both 1D NMR and CPMG as there was little variation between the ENT of the different analyses. The ENT implicitly quantifies the level of dependency within the data. Features (individual ppms) that were significantly (MWSL) associated with either CAC or IMT in MESA were annotated (as described in the metabolite identification section, above) and within each cluster we selected the ‘sentinel’ feature with the lowest *P*-value. In tables, we present the cluster with the lowest *P*-value per metabolite, as more than one cluster may correspond to the same metabolite. We then took these features forward for replication in the Rotterdam Study and LOLIPOP using regression models with adjustment for the same confounders, and further adjusting for cohort. Features with *P* <0.05 for the same outcome of interest and with a consistent direction of association as in MESA were considered replicated. We considered replicated metabolites our principal finding (as reported in our main Figures and Abstract) and also performed additional investigations, as detailed below.

#### Additional analyses

We examined in the three cohorts combined the association between replicated features and atherosclerosis (separately for CAC and IMT) adjusting also for the main cardiovascular risk factors [LDL and HDL cholesterol, systolic blood pressure, smoking status (current, past, and never smoker), and diabetes) as well as lipid and blood pressure lowering treatment (Model 2). We also examined associations between replicated features and incident cardiovascular events (MI and Stroke) in MESA and the Rotterdam Study using Cox regression with adjustment for the same set of confounders as in Model 2 above and with duration of follow-up as the time metric. Proportional hazards assumptions were tested using Schoenfeld residuals implemented in R Package Survival and risk estimates were presented as hazard ratio (incident event data were unavailable in the LOLIPOP study). For metabolites that were significant at MWSL level in Model 2, we performed stepwise linear regression for CAC and IMT separately in MESA with all Model 2 confounders forced in the model as implemented in the R package ‘bootStepAIC’^28^ on 1000 bootstrap samples. Results are summarized by counting how many times each variable is selected and how many times the estimate of the regression coefficient of each variable changed signs. We performed analyses on the Bruker lipoprotein data for MESA samples, using linear regression models with adjustment for Model 2 confounders except for blood lipids (LDL and HDL cholesterol) (Model 3: age, sex, ethnicity, phase, systolic blood pressure, smoking status, and diabetes, lipid and blood pressure lowering treatment). Principal component analysis of the 105 lipoproteins concentration showed that 10 principal components explained more than 95% of the total variation in the data set. Therefore, the Bonferroni corrected significance level accounting for the correlation in the data was *P* = 0.05/10 (*P* = 0.005) in these analyses.

Finally, in MESA, we examined associations between replicated metabolic features and cardiometabolic risk factors [body mass index (BMI), waist circumference, systolic blood pressure, diabetes, LDL and HDL cholesterol, and inflammatory biomarkers] using linear regression adjusted for model two confounders. Results are presented via a correlation matrix.

We performed sensitivity analyses excluding individuals with diabetes treatment (*N* = 765) or blood pressure treatment (*N* = 2603) or lipid lowering treatment (*N* = 1196) and finally excluding individuals with self-reported diabetes or on diabetes treatment (*N* = 1060). We also examined models with adjustment for BMI in addition to cardiovascular risk factors included in Model 2.

All analyses were performed using the R project software (‘Development Core Team, R’, 2005) using packages rockchalk to estimate the partial correlation^29^ and openxlsx to write results file^30^ as well as multiple helper functions to manipulate and visualize data.^31–34^

### Metabolic reaction network

The Metabonetwork method^35^ was applied in Matlab [Version 8.3 (R2014a), the Mathworks Inc., Natick, MA, USA] to create a sub-network of metabolic reactions associated with CAC and IMT. The algorithm identifies the main reaction pairs as defined in the Kyoto Encyclopaedia of Genes and Genomes (KEGG)^36^ occurring spontaneously or by the activity of an enzyme related to *Homo sapiens* genes and calculates the adjacency matrix for all compounds based on these main reaction pairs. The shortest metabolic paths between all metabolites significantly associated with IMT and CAC were then determined based on the adjacency matrix and a network graph was drawn to display the selected metabolites and all connected compounds with the shortest paths.

### Ingenuity pathway analysis

We used Ingenuity Pathway Analysis (IPA) to investigate the gene and metabolite networks linked to the ^1^H NMR-derived metabolites of atherosclerosis.^37^ First, we uploaded the list of CAC-IMT associated metabolites into IPA, considering only experimentally observed molecular relationships with the maximum number of molecules per generated molecular network included as the default. IPA generated a shortlist of interaction networks around the metabolites of interest. These were then merged into a single combined network.

## Results

*Table 1* shows the baseline characteristics across the three studies and Supplementary material online, *Figure S6* shows the flowchart of participants in this study.

**Table 1:** Numbers of individuals, and means (standard deviation) or percent for demographic, anthropometric and clinical outcome variables

	LOLIPOP	Rotterdam	MESA	All
*N*	1917	1652	3867	7436
Gender (female/male)	662/1255	887/765	1957/1910	3506/3930
Age (years)	54.8 (10)	70.8 (5.7)	62.9 (10.3)	62.6 (10.9)
Body mass index (kg/m^2^)	27.4 (4.4)	27 (3.9)	28.1 (5.4)	27.7 (4.9)
Coronary artery calcium (CAC) >0 (%)	1011 (52.7)	1491 (90.3)	2016 (52.1)	4518 (60.8)
CAC (Agatston score)	154.1 (439.6)	486.6 (915.6)	155.3 (423.6)	228.6 (590.2)
Coronary artery calcium (log(CAC + 1))	2.2 (2.5)	4.4 (2.4)	2.3 (2.6)	2.8 (2.7)
Intima-media thickness (IMT) (mm)	0.7 (0.1)	1.1 (0.2)	0.8 (0.2)	0.8 (0.2)
Intima-media thickness (log_10_(IMT))	−0.2 (0.1)	0 (0.1)	−0.1 (0.1)	−0.1 (0.1)
Systolic blood pressure (mmHg)	131.3 (18.9)	143.1 (21.2)	127 (21.4)	131.7 (21.7)
Total cholesterol (mg/dL)	209.9 (40.4)	224.5 (37)	194 (35)	204.9 (38.9)
HDL cholesterol (mg/dL)	52.3 (13.5)	53.8 (14.9)	50.9 (14.7)	51.9 (14.5)
LDL cholesterol (mg/dL)	130.9 (34.6)	144.2 (33.6)	117.3 (31.4)	126.8 (34.5)
Diabetes (%)	287/1630 (15)	236/1416 (14.3)	537/3330 (13.9)	1060/6376 (14.3)
Current smoker (%)	205/1712 (10.7)	284/1368 (17.2)	467/3400 (12.1)	956/6480 (12.9)
Ethnicity (%)	813 (42.4%) Caucasian	1652 (100%) Caucasian	1492 (38.6%) Caucasian	3952 (53.2%) Caucasian
	1104 (57.6%) Indian		521 (13.5%) Asian	1104 (14.8%) Indian
			949 (24.5%) African	521 (7%) Asian
			905 (23.4%) Spanish	949 (12.8%) African
				905 (12.2%) Spanish
Lipid lowering treatment (yes/no) (%)	303/1614 (15.8)	252/1400 (15.3)	641/3226 (16.6)	1196/6240 (16.1)
Blood pressure treatment (yes/no) (%)	494/1423 (25.8)	646/1006 (39.1)	1463/2404 (37.8)	2603/4833 (35)
Diabetes treatment (yes/no) (%)	287/1630 (17.6)	97/1555 (6.2)	381/3486 (10.9)	765/6671 (11.5)
Incident cardiovascular events (yes/no) (%)	—	379/1093 (16)	251/3613 (6.5)	487/4849 (9.1)

### Nuclear magnetic resonance metabolomics and atherosclerosis

**Figure 1:**
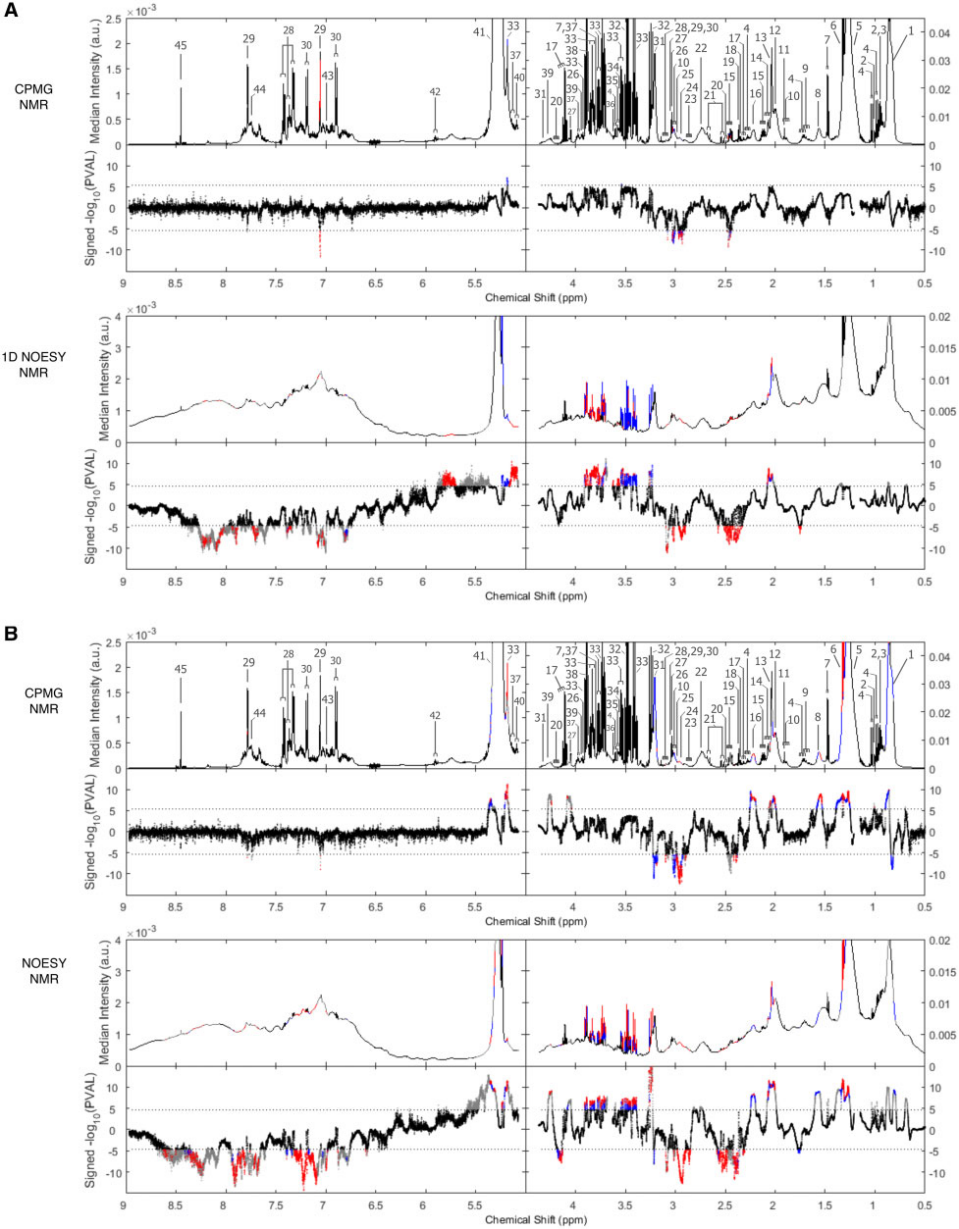
Manhattan-type plot showing the analysis of the 30 590 Carr-Purcell-Meiboom-Gill nuclear magnetic resonance (upper panel) and one-dimensional nuclear magnetic resonance (lower panel) features with (*A*) coronary artery calcium and (*B*) intima-media thickness in minimal adjusted model (Model 1: age, sex, cohort, and ethnicity) and fully adjusted model (Model 2: low and high-density lipoproteins, lipid and blood pressure lowering treatment, systolic blood pressure, smoking status, and diabetes). The signed −log10 *P*-value (on the *y*-axis) is derived from Model 1. The black dots represent the data points remaining significant after multiple testing correction (1.8 × 10^−5^ for one-dimensional nuclear magnetic resonance and 3.7 × 10^−6^ for Carr-Purcell-Meiboom-Gill nuclear magnetic resonance, respectively), the blue dots are Model 1 significant in MESA and replicated in the Rotterdam Study and LOLIPOP (with a *P*-value <0.05), and the red dots are Model 2 significant. The horizontal axis is the nuclear magnetic resonance chemical shift (in ppm). Median spectra intensity is given in the pooled dataset non-missing for coronary artery calcium/intima-media thickness together with chemical compounds. Nuclear magnetic resonance assignments: 1, lipids (low-density lipoprotein and very low-density lipoprotein, **CH3**-CH2-R, **CH3**-CH2-C=); 2, isoleucine; 3, leucine; 4, valine; 5, lipids (low-density lipoprotein and very low-density lipoprotein, CH3-**CH2**-R, (**CH2**)n); 6, lactate; 7, alanine; 8, lipids (low-density lipoprotein and very low-density lipoprotein, **CH2**-CH2-C=, **CH2**-CH2-CO); 9, arginine; 10, lysine; 11, acetate; 12, lipids (CH2-**CH2**-CH=CH); 13, N-acetylglycoproteins; 14, methionine; 15, glutamine; 16, lipids (**CH2**-CO); 17, 3-hydroxybutyrate; 18, glutamate; 19, pyruvate; 20, 5-oxoproline; 21, citrate; 22, lipids (CH=CH-**CH2**-CH=CH); 23, aspartate; 24, albumin; 25, N,N-dimethylglycine; 26, creatine; 27, creatinine; 28, phenylalanine; 29, histidine; 30, tyrosine; 31, choline; 32, beta-glucose; 33, beta-glucose; 34, glycine; 35, glycerol; 36, myo-inositol; 37, mannose; 38, 1,5-anhydrosorbitol; 39, glyceryl groups of lipids; 40, acetaminophen+ glucuronide; 41, lipids (**CH=CH**); 42, uridine; 43, 1-methyl histidine; 44, 3-methylhistidine; and 45, formate.

We analysed the full resolution metabolic profiles^27^ from standard 1D NMR and CPMG NMR spectra in relation to CAC and IMT.^16^ For 1D NMR, 7935 spectral features, corresponding to 74 NMR spectral regions (clusters, see Methods section) were associated with CAC in MESA (*P *<* *1.8 × 10^−^^5^) and of them 41 regions were replicated (*P *<* *0.05) in samples from Rotterdam Study and LOLIPOP (*Figure 1A*, Supplementary material online, *Table S2*). From these features, 19 metabolites were annotated (Supplementary material online, *Table S2*); we were unable to annotate two regions which corresponded to broad signals present in the spectral baseline, likely corresponding to multiple overlapping resonances from serum proteins (see Methods and Supplementary material online, *Figures S2–S5*). Overall 10 metabolites were directly associated with CAC (alanine, glycine, methionine, glucose, acetaminophen-glucuronide, glycerol, acetyl glycoproteins, *myo*-inositol, mannose, and 1,5-anhydrosorbitol) and 9 were inversely associated (glutamate, glutamine, N,N-dimethylglycine, lysine, phenylalanine, 5-oxoproline, 3-hydroxybutyrate, citrate, and albumin).

In discovery analyses, where IMT was used instead of CAC as the marker of atherosclerosis, 22 annotated metabolites were replicated in the analysis of the pooled Rotterdam Study and LOLIPOP samples including 1-methylhistidine, 3-hydroxybutyrate, aspartate, and tyrosine (inverse), and lactate, and valine (direct) (*Figure 1B*, Supplementary material online, *Table S3*). Of these 22 metabolites, 12 were replicated metabolites associated with CAC, while 10 were only associated with IMT but not CAC.

In analyses of CPMG NMR data, we identified five additional metabolites associated with CAC and IMT (Supplementary material online, *Tables S2* and *S3*).

### Adjustment for cardiovascular disease risk factors

After further adjustment for CVD risk factors, the magnitude of the aforementioned association between the metabolites and atherosclerosis attenuated by at least 50%. In detail, mannose, alanine, and acetaminophen-glucuronide showed strong direct associations and glutamate and histidine strong inverse associations with CAC below the MWAS significance threshold (*P *=* *2.4 × 10^−^^9^ to 1.1 × 10^−^^5^); all other replicated metabolites remained nominal statistically significant at *P *<* *0.05. In analyses where IMT was the marker of atherosclerosis, histidine, N,N-dimethylglycine, and albumin showed strong inverse associations below the MWSL threshold (*P *=* *7 × 10^−^^7^ to 6.7 × 10^−^^6^); 14 other metabolites retained nominal statistical significance (*P *<* *0.05). Only histidine was associated with both CAC and IMT at MWSL significance level after adjustment for CVD risk factors. In stepwise linear regression with CVD risk factors, acetaminophen glucuronide and histidine remained in the model for CAC and histidine and albumin for IMT (Supplementary material online, *Figure S7*). In sensitivity analyses, exclusion of people receiving treatment for blood pressure, diabetes or lipids, and analyses adjusting for BMI in addition to CVD risk factors did not materially affect these results (Supplementary material online, *Tables S4*–*S8*).

### Metabolites associated with incident cardiovascular disease

**Figure 2:**
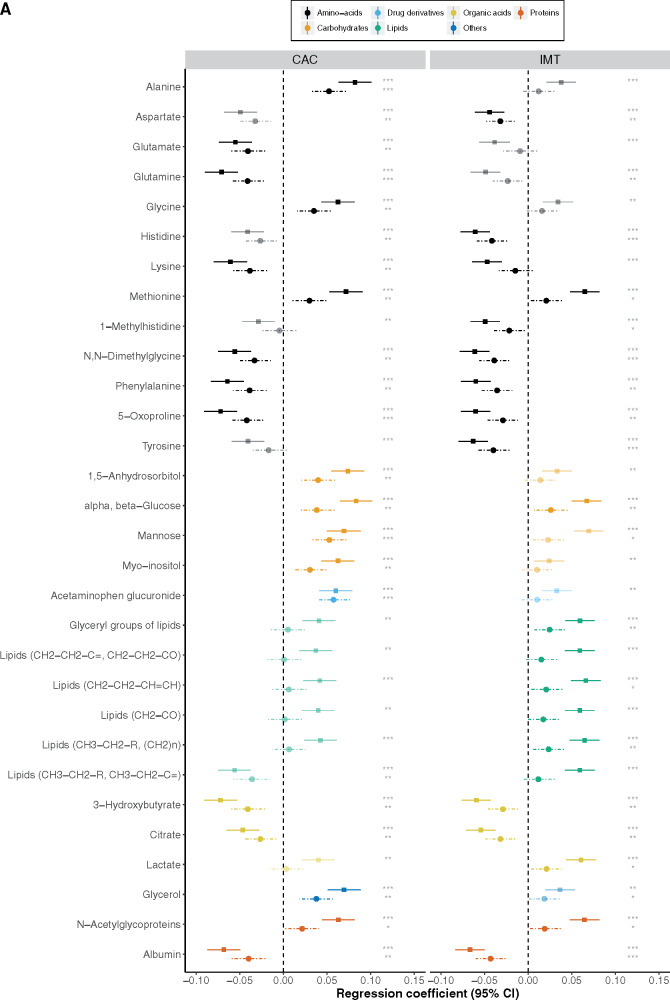

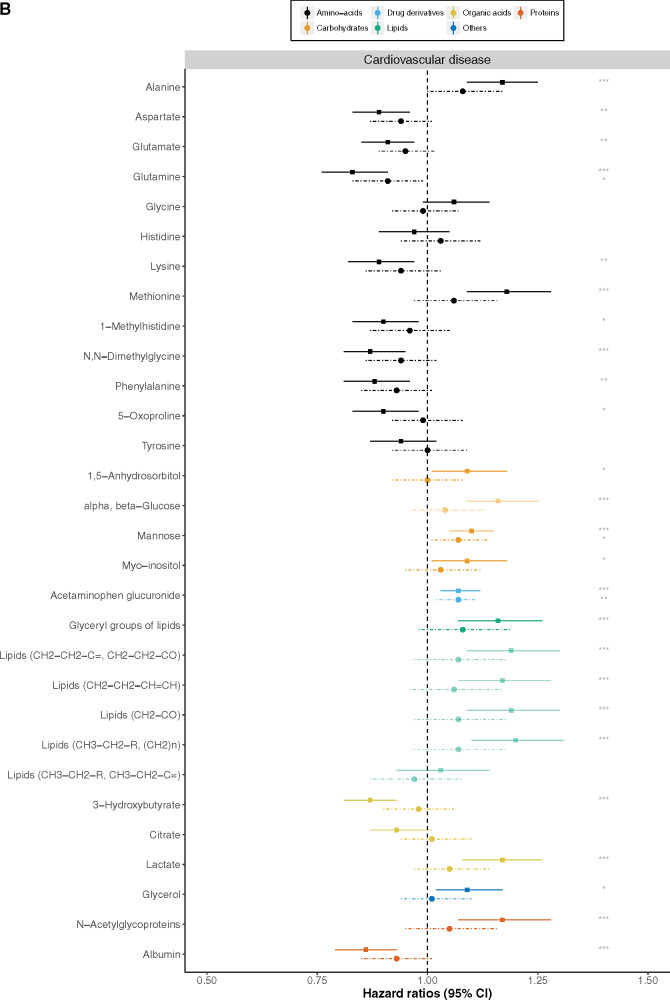
(*A*) Regression coefficients per standard deviation (95% confidence interval) between one-dimensional nuclear magnetic resonance metabolites associated with coronary artery calcium and/or intima-media thickness using the sentinel (most significant) ppm within each nuclear magnetic resonance region (cluster) pooled across all three cohorts (*N* = 7436) and coronary artery calcium and intima-media thickness. The solid lines represent Model 1 (adjusted for age, sex, cohort, and ethnicity) and the dotted lines Model 2 (further adjusted for low-density lipoprotein and high-density lipoprotein, lipid and blood pressure lowering treatment, systolic blood pressure, smoking status, and diabetes). The significance threshold is given for Models 1 and 2 where *P ≤* *0.05, **P ≤* *0.01, and ***P ≤* *1.8e^−05^ (metabolome wide significance level for one-dimensional nuclear magnetic resonance data, see Supplementary material online, *Table S1*). (*B*) Hazard ratios (95% confidence interval) per standard deviation between the one-dimensional nuclear magnetic resonance metabolites associated with coronary artery calcium and/or intima-media thickness and incident cardiovascular disease events in MESA and Rotterdam studies (*N* = 630 events). The significance threshold is given for Models 1 and 2 where *P ≤* *0.05, **P ≤* *0.01, and *** *P *≤* *0.001.

*Figure 2A* shows the 1D NMR metabolites that were replicated in their associations with either CAC or IMT. There was considerable overlap and consistency in the metabolic signature between the two phenotypes with all metabolites showing the same direction of association between the atherosclerotic measures. The majority of metabolites that were associated with measures of atherosclerosis were also associated with incident CVD events available in MESA and Rotterdam Study participants (*Figure 2B*, Supplementary material online, *Table S9*) with 25 out of 30 metabolites reaching nominal statistical significance (*P* < 0.05). However, adjustment for CVD risk factors attenuated all associations markedly with only acetaminophen glucuronide showing a *P*-value smaller than 0.05.

### Lipoproteins associated with atherosclerosis and incident cardiovascular disease

**Figure 3:**
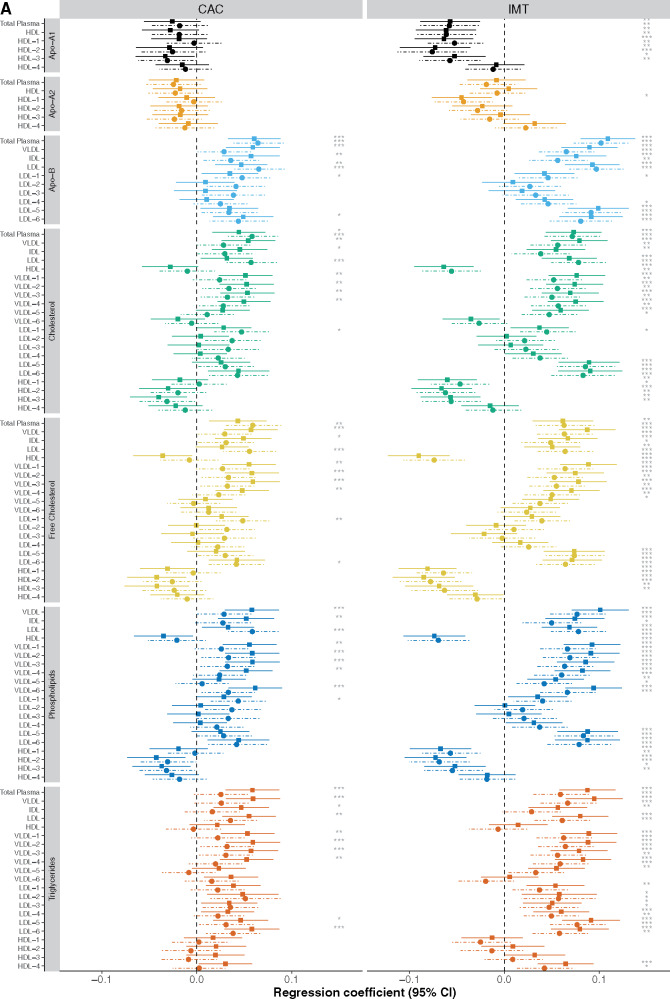

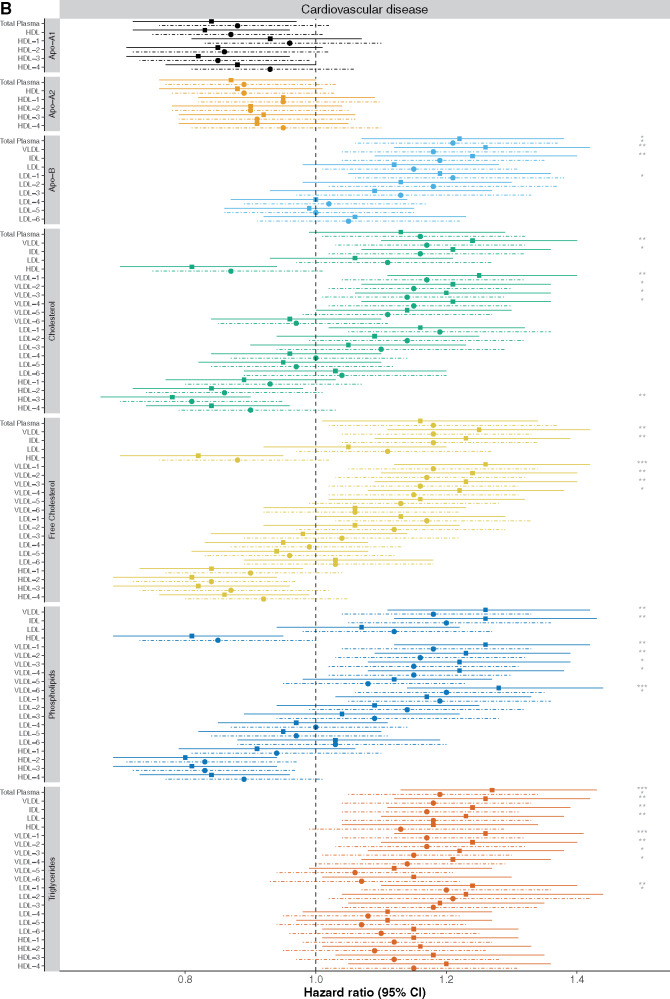
Associations between lipoprotein particles from Bruker analysis in MESA (*N* = 3753). The solid lines represent Model 1 and the dashed lined Model 3. (*A*) The regression coefficient (95% confidence interval) per standard deviation of each lipoprotein between coronary artery calcium and each nuclear magnetic resonance lipoprotein feature was adjusted for age, sex, ethnicity, and analysis phase (Model 1) and further adjusted for basic cardiovascular risk factors (diabetes, systolic blood pressure, smoking and medication for hypercholesterolaemia, diabetes or high blood pressure) (Model 3). (*B*) Hazard ratios (95% confidence interval) for incident cardiovascular disease events (*N* = 242) and lipoproteins are shown. A significance threshold with adjusted Bonferroni correction is given for Models 1 and 2 where *P_adj_ ≤0.005, **P_adj_ ≤0.001, and ***P_adj_≤ 0.0001. The *P*-value Bonferroni corrected by the number of PCs (10) that account for more than 95% of the total variation in the data set. A *P *<* *0.05/10 (<0.005) was therefore used to denote statistical significance in these analyses (see Methods section). Analysis of 105 lipoprotein subclasses was carried out including different chemical components of intermediate-density lipoprotein (density 1.006–1.019 kg/L), very low-density lipoprotein (0.950–1.006 kg/L), low-density lipoprotein (density 1.09–1.63 kg/L), and high-density lipoprotein (density 1.063–1.210 kg/L). The low-density lipoprotein sub-fraction was fractionated into six density classes (low-density lipoprotein-1 1.019–1.031 kg/L, low-density lipoprotein-2 1.031–1.034 kg/L, low-density lipoprotein-3 1.034–1.037 kg/L, low-density lipoprotein-4 1.037–1.040 kg/L, low-density lipoprotein-5 1.040–1.044 kg/L, low-density lipoprotein-6 1.044–1.063 kg/L), and the high-density lipoprotein sub-fraction in four density classes (high-density lipoprotein-1 1.063–1.100 kg/L, high-density lipoprotein-2 1.100–1.125 kg/L, high-density lipoprotein-3 1.125–1.175 kg/L, and high-density lipoprotein-4 1.175–1.210 kg/L).

To reveal more detailed information on lipoprotein content, we used the Bruker Lipoprotein Subclass Analysis to deconvolve two specific 1D NMR signals corresponding to the lipid moiety C**H_3_**-C**H_2_**-R in MESA data (see Methods section). Overall, 35/105 lipoprotein subclasses were significantly (*P *<* *0.005) associated with CAC and three of them (total plasma cholesterol, total plasma apolipoprotein B, and apolipoprotein B within total plasma LDL) retained statistical significance (*P *<* *0.005) after adjustment for non-lipid CVD risk factors (*Figure 3*, Supplementary material online, *Table S10*). For IMT, associations with lipids were stronger and 74/105 and 63/105 lipoproteins showed significant (*P *<* *0.005) associations in minimal and fully adjusted models, respectively (*Figure 3*, Supplementary material online, *Table S11*). The pattern of the association between lipoproteins and IMT and CAC was again consistent between the two measurements (*Figure 3*, Supplementary material online, *Tables S11* and *S12*). Lipoproteins were also associated with incident CVD with apolipoprotein B and free cholesterol in very low-density lipoproteins showing the strongest associations and overall lower density lipoproteins showing stronger direct associations with atherosclerosis and CVD events (*Figure 3*, Supplementary material online, *Table S12*). Triglycerides in all measured major lipoprotein subclasses including HDL were positively associated with higher risks of CVD.

### Metabolic pathways

**Figure 4:**
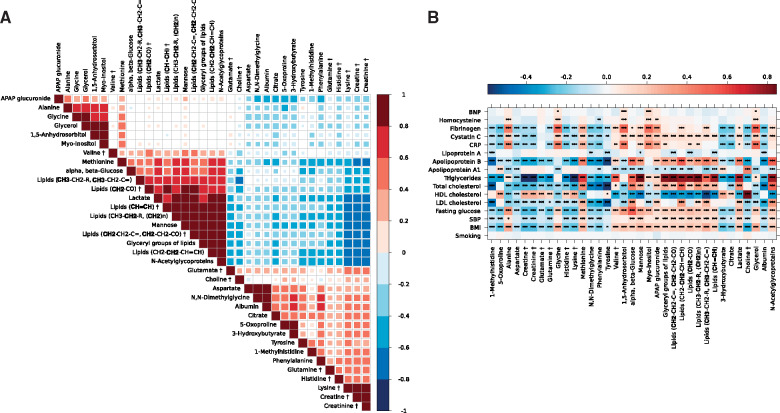
(*A*) Partial correlations between markers of coronary artery calcium or intima-media thickness in MESA (*N* = 3948), using sentinel ppm for each one-dimensional or Carr-Purcell-Meiboom-Gill assigned metabolite (*N* = 35). ^†^Metabolite assessed in Carr-Purcell-Meiboom-Gill data. Adjusted analysis controlling for sex, age, ethnicity, and measurement phase. ***The threshold after Bonferroni correction for 560 tests ensuring a Family-Wise Error Rate control at 0.1% (0.001/560), ** at 1% (0.01/560), and * at 5% (0.05/560). (*B*) Spearman correlation matrix between metabolites associated with coronary artery calcium and/or intima-media thickness (*N* = 35) and cardiovascular disease risk factors, using the sentinel (most significant) ppm within each nuclear magnetic resonance region (cluster) in MESA (*N* = 3948) with colour-keyed correlation coefficient. Hierarchical clustering was used to reorder the correlation matrix. The size of the squares is proportional to the significance level; statistical significance was set to a Bonferroni threshold correction ensuring a Family-Wise Error Rate control at 5%. ^†^Metabolite detected in the Carr-Purcell-Meiboom-Gill data.

**Figure 5:**
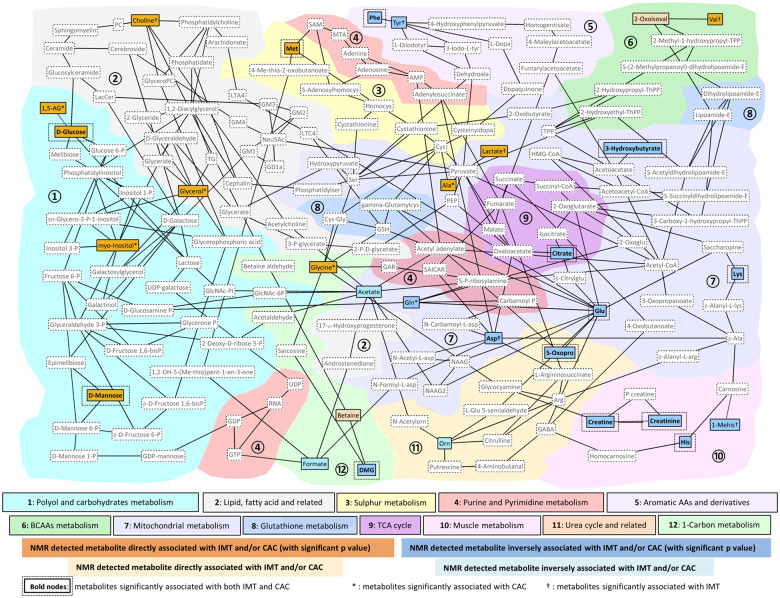
Multicompartmental metabolic network characterizing subclinical atherosclerosis. Metabolites highlighted in strong colours passed the multiple testing correction in MESA (1.8 × 10^−5^) and were replicated in independent populations (the Rotterdam Study and LOLIPOP) for Model 1 (adjusted for age, sex, cohort, and ethnicity) in relation to coronary artery calcium and/or intima-media thickness. Nodes and edges in the graph represent metabolites and reactions from the Kyoto Encyclopaedia of Genes and Genomes. Metabolites from Kyoto Encyclopaedia of Genes and Genomes are included in the ‘metabonetwork’^25^ if they were present on the set of shortest paths between the metabolites associated with coronary artery calcium/intima-media thickness. The direction of association between metabolites and coronary artery calcium/intima-media thickness is illustrated in the graph by the orange (direct) and blue (inverse) colours, and was consistent within each pathway. Full names of abbreviations are listed in the Supplementary material online, *Table S14*.

Correlations between the metabolites revealed a strong dependence structure between the replicated metabolic signals (*Figure 4A*). Metabolite markers of atherosclerosis and CVD risk factors, including blood pressure and lipids, were strongly associated in the same direction as the associations with CAC and IMT (*Figure 4B*). The human metabolic network from KEGG was used to compute the shortest metabolic paths between metabolite markers of atherosclerosis, and plot their connectivities in a network graph (a so-called ‘metabonetwork’,^25^*Figure 5*). The figure illustrates disturbances of pathways including those related to lipid, fatty acid and carbohydrate metabolism, branched chain amino acid (BCAA) and aromatic acid metabolism, tricarboxylic acid (TCA), and urea cycle and muscle metabolism. We further investigated gene and metabolite networks by integrating our results with previously experimentally observed molecular relationships using Ingenuity Pathway Analysis (IPA, Supplementary material online, *Figure S8*). IPA revealed an interaction network between genes mainly involved in inflammatory, insulin and lipid pathways, and our ^1^H NMR-identified metabolite markers of atherosclerosis.

**Take home figure:**
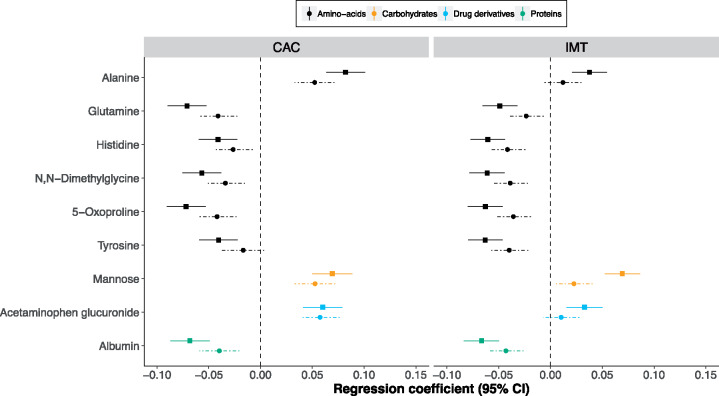
Metabolites associated at metabolome-wide significant level with at least one measure of atherosclerosis assessed via coronary artery calcium or intima-media thickness before (solid lines) and after (dotted lines) adjustment for conventional cardiovascular risk factors.

## Discussion

In this study of over 7000 participants from three prospective population-based cohorts, we present the metabolic signature of atherosclerosis offering insights into the widespread systemic disturbances underlying atherosclerosis. Atherosclerosis was associated with disturbances of inter-connected pathways related to lipid, fatty acid and carbohydrate metabolism, BCAA and aromatic acid metabolism, TCA and urea cycle, and muscle metabolism and showed a largely consistent pattern between coronary and carotid atherosclerosis. Subsequently, these metabolites were also associated with incident CVD events highlighting the importance of these pathways in progression to clinical CVD. The fact that the majority of these associations attenuated substantially after adjustment for conventional CVD risk factors suggests that these metabolites lie on pathways closely associated with CVD risk factors.

Beyond KEGG pathways, replicated metabolites highlight oxidative stress and inflammatory pathways. For example, 5-oxoproline and glutamate, both inversely associated with CAC and IMT, are involved in both synthesis and degradation of glutathione. Glutathione deficiency contributes to oxidative stress, which plays a key role in the pathogenesis of atherosclerosis.^38^^,^^39^ N-acetylneuraminic acid, positively associated with CAC and IMT, is the major form of sialic acid in mammals and serves as a biomarker of a sustained inflammatory response with subsequent effects on atherosclerosis.^39^^,^^40^ Glycoprotein acetyls have previously shown associations with risk of MI and stroke,^41^ and here, we also report consistent effects with CAC and IMT. In addition, lactate, also associated positively with IMT, formed from pyruvate under insufficient oxygen supply (hypoxia), is an indicator of inflammation. Local hypoxia can occur in highly active inflamed tissues where demands from increased cellularity exceed oxygen supply, a feature of atherosclerotic plaque.^42^ Hypoxia has been hypothesized to stimulate pro-atherosclerotic processes, including deficient lipid efflux, inflammation, interference with macrophage polarization and glucose metabolism.^43^

The four metabolites linked to sugar and carbohydrate metabolism (D-glucose, 1,5-anhydrosorbitol, D-mannose, and myo-inositol) were all directly associated with both CAC and/or IMT after adjustment for CVD risk factors. 1,5-anhydrosorbitol, a marker of glycaemic control, has previously been associated with CVD and kidney disease and highlights the close association of atherosclerotic disease, diabetes and insulin resistance.^38^^,^^44^^,^^45^ Mannose has repeatedly been associated with pre-diabetes, incident Type 2 diabetes,^46^ and all-cause mortality,^47^ and here, we highlight associations with atherosclerosis and incident CVD, independent of glucose. In this regard, mannose showed stronger correlations with lipids and N-acetylglycoproteins than with glucose (*Figure 4*). Mannose has a central role in glycation processes of lipoproteins that in turn may play a role in the initiation and development of atherogenesis. N-glycans are up-regulated in pro-inflammatory settings and have been found on the endothelial cell surface in early stages of atherosclerotic plaque development.^48^ Taken together these data suggest that mannose could also affect CVD risk through non-glucose dependent pathways.

Energy metabolism, TCA cycle and glycolysis, were also central pathways associated with atherosclerosis. Alanine is synthesized directly from pyruvate, a product of glycolysis, which can supply the cells with energy through the TCA cycle in aerobic conditions. Increased glucose utilization has been shown in high-risk atherosclerotic plaques.^49^ In turn, citrate is an intermediate and key energy metabolite in the TCA cycle and has previously been associated with cardiovascular mortality.^50^ Reduced oxygen levels in presence of atherosclerosis may affect the TCA cycle since it is oxygen dependent. Creatine, an amino acid derivative, reflects changes of energy metabolism in the muscles; it is transported through the circulation and taken up by tissues with high energy demands, with creatinine as a degradation product. Their levels have been inversely associated with fat intake in animal studies.^51^^,^^52^

The lipoprotein profiles analyses confirmed previous observations investigating the associations of these lipids with subclinical atherosclerosis,^53^ MI, and stroke.^41^ Here, we further show a largely consistent picture between coronary and carotid atherosclerosis and with cardiovascular events. Association of triglycerides with atherosclerotic disease and future events was positive even within HDL particles in support of previous observations. Conversely, not all HDL cholesterol was inversely associated with atherosclerosis highlighting the fact that causal association of HDL with lower CVD risk may be limited to certain particles (i.e. not the ones containing triglycerides). In contrast, LDL and VLDL was consistently positively associated with higher CAC and IMT and higher risk of CVD events with a small trend showing stronger associations with decreasing density of lipoproteins.

The molecular interaction gene network map (Supplementary material online, *Figure S6*) further depicts the interconnections between inflammatory, insulin, and lipid pathways reflected by metabolite markers of atherosclerosis. For example, the network includes fibronectin 1 (FN1), which is involved in cell adhesion and migration processes including wound healing, blood coagulation, and host defence; genetic polymorphisms within this gene have been associated with adverse lipid levels and coronary heart disease.^54^^,^^55^ Other examples include CCAAT/enhancer-binding protein beta, a master regulator of immunity and inflammation, other immunity related genes (e.g. *PI3K* complex), and pathways related to betaine-homocysteine metabolism (*BHMT*), glycolysis (pyruvate kinase), and angiogenesis (*ANGPT4*).

Our study has several strengths. To our knowledge, it is the largest to investigate the metabolic signature of atherosclerosis using both standard 1D and CPMG ^1^H NMR spectra with information on small molecules and lipoprotein subclasses. We replicated our findings in external independent populations and we used two phenotypes of atherosclerosis (CAC and IMT) to further increase the generalizability and robustness of our findings. We characterized the underlying biological pathways associated with the metabolites of interest, highlighting extensive inter-connected disturbances of metabolism.

One limitation of our study is the cross-sectional nature of associations of metabolites with sub-clinical atherosclerosis; however, we also examined our results in relation to prospective data using incident CVD events. Nonetheless, as with any observational study, causality cannot be inferred and further experimental and functional work is needed to further elucidate the metabolic pathways involved in atherosclerotic disease. We studied metabolites present in blood which may not accurately reflect metabolic processes in atherosclerotic plaques and arterial tissue, although there is strong evidence to suggest that at least some blood biomarkers (e.g. cholesterol) are relevant to atherosclerosis and blood is a readily accessible tissue for identification of prognostic or diagnostic markers. Finally, since we used a untargeted metabolic profiling approach, some of the discovered metabolic features could not be annotated (as expected); however, most of the unannotated signatures correspond to broad signals most likely from protein residues which cannot be further annotated by NMR. At the same time, this untargeted (‘agnostic’) approach allows for the discovery of metabolites that are not part of targeted panels. Our analysis was limited to NMR and mass spectrometry data may offer additional insights not apparent based on NMR data alone.

In summary, in this study of over 7000 participants from three prospective population-based cohorts, we found strong associations between serum metabolites observed on ^1^H NMR spectroscopy and subclinical atherosclerosis which was largely consistent between the two vascular beds (coronary and carotid arteries). Our metabolic and gene networks reveal highly inter-connected system level metabolic disturbances in atherosclerosis, much of which overlaps with the known cardiovascular risk factors. Genetic approaches as well as mechanistic studies are now needed to further validate our results and to follow-up the possible entry points for investigation of novel targets or preventive strategies for atherosclerotic disease.

## Supplementary Material

ehz235_Supplementary_Data

## Notes

**See page 2897 for the editorial comment on this article (doi: 10.1093/eurheartj/ehz252)**
