# Supplementary material for: Serum metabolic signatures of coronary and carotid atherosclerosis and subsequent cardiovascular disease
Source: Eur Heart J. 2019 May 18;40(34):2883–96. doi: 10.1093/eurheartj/ehz235 (PMC7963131; doi:10.1093/eurheartj/ehz235)

Supplementary Material Online

Serum metabolic signatures of coronary and carotid atherosclerosis and subsequent cardiovascular disease

Ioanna Tzoulaki[1,2,3,4*+], Raphaële Castagné[1,5*], Claire L Boulangé[6,7*], Ibrahim Karaman[1,2], Elena Chekmeneva[7], Evangelos Evangelou[1,2,3], Timothy M. D. Ebbels[7], Manuja R. Kaluarachchi[6,7], Marc Chadeau-Hyam[1,2], David Mosen[1,2], Abbas Dehghan[1,2], Alireza Moayyeri[8], Diana L. Santos Ferreira[9], Xiuqing Guo[10], Jerome I. Rotter[10], Kent D. Taylor[10], Maryam Kavousi[11], Paul S. de Vries[11,12], Benjamin Lehne[1], Marie Loh[1], Albert Hofman[11,13], Jeremy K. Nicholson[6,7], John Chambers[1,14], Christian Gieger[15], Elaine Holmes[6,7], Russell Tracy[16], Jaspal Kooner[14,17], Philip Greenland[18], Oscar H. Franco[11,19], David Herrington[20*], John C. Lindon[6,7*], Paul Elliott[1,2,4*+]

* These authors contributed equally

Data Supplement

1H NMR spectroscopy: sample preparation and data acquisition.

The NMR spectra were measured in two phases, labelled phase 1 and phase 2. Quality control samples were included in the NMR analyses to monitor the system reproducibility. The QC samples in phase 1 were prepared from a commercial serum sample (Sigma). In phase 2, the same QC samples were included in the analysis and a set of pooled phase 1 samples from the LOLIPOP cohort was also used for QC purposes. Multiple aliquots of QC samples were stored in -80°C prior to the NMR analysis.

Frozen serum and QC samples (-80°C) were thawed and centrifuged at 2700 × g for 10 min to remove precipitated proteins and other particles. A volume of 300 µl from the supernatant was mixed with 300 µl of phosphate buffer (0.142 M Na_2HPHO_3, pH=7.4, 4% sodium azide and 0.08% trimethylsilyl propionic acid (TSP)) in an Eppendorf tube and transferred into a 5mm diameter NMR tube. During the analysis, the samples were maintained at 4°C in a NMR Sample Jet (Bruker Biospin). ^{1}H standard 1D (NOESYPR1D) NMR spectra with water pre-saturation and ^{1}H CPMG NMR spectra were recorded on a 600 MHz Avance III Bruker NMR spectrometer (Bruker Biospin, Rheinstetten, Germany) operating at 600.13 MHz. All of the experiments were carried out at a temperature of 310K. For the NOESYPRESAT NMR spectra the standard pulse sequence with insertion of magnetic field gradients in the mixing period was used (Bruker Biospin). The time between the first and second 90° RF pulses was 3 µs, and the mixing time was 10 ms. A relaxation delay of 4 s was used between each FID acquisition.

For the CPMG NMR spectra, the following parameters were used: a spin echo delay of 0.3 ms and 128 loops resulting in a total T2 relaxation delay of 76.8 ms. A relaxation delay of 4 s between 2 FIDs was also applied. For both CPMG and NOESYPRESAT NMR analyses, 32 scans were acquired into 96,000 data points. All FIDS were multiplied by an exponential function equal to a line broadening factor of 0.3 Hz, zero filled to 128k and then Fourier transformed. All spectra were phase- and baseline corrected automatically using Bruker software (TopSpin 3.2, Bruker Biospin). The chemical shifts were referenced to that of the anomeric H1 proton of α-glucose taken to be at 5.233 ppm from TSP.

1H NMR processing steps across 3 cohorts.

The processing workflow to integrate the multi-cohort ^{1}H NMR metabolomics data has been described (Karaman et al 2016). In summary:

1. The data tables were concatenated in order to have one large data table consisting of ~8,000 samples and 34,001 variables (δ 0.500–9.000).
2. The region δ 4.400–5.100 corresponding to H_2O resonances was removed and the data table was divided into six slices because of the computational issues due to high computer memory demand. The ends of the slices were selected from the noisy regions.
3. Spectral peak alignment on each slice was performed by Recursive Segment-wise Peak Alignment (RSPA) algorithm (Veselkov et al., 2009) and thereafter the slices were concatenated back. In order to evaluate spectral peak alignment, alignment quality measures (aq_{bin}) were calculated and compared for bin sizes of δ = 0.02 and 0.08 ppm for unaligned and aligned data (Veselkov et al., 2009). Higher values for $aq_{0.02}$ and $aq_{0.08}$ in the aligned data compared to the unaligned data were obtained proving a successful spectral peak alignment.
4. Selected regions such as δ 1.180–1.240, δ 2.244–2.261, δ 3.375–3.400 and δ 3.660–3.710 where the peaks of different suspected contaminations occur were removed from the whole spectra.
5. The spectra with the remaining regions were normalised by probabilistic quotient normalisation using the median spectrum as the reference (Dieterle et al., 2006).

Assessment of pre-processing was achieved by monitoring the quality control samples among all the samples on the score plots of the first few components resulting in principal component analysis of the data table. Pre-processing improved clustering of the quality control samples, therefore the data became ready for further statistical analysis. Furthermore, extreme outlier samples were examined using the same score plots and removed. Excluded samples were attributable to spectra with poor water suppression and baseline distortion. The results for the assessment of the data quality are presented in [ref].

As the last processing step, The variation between six data tables due to differences between the cohorts and phases was removed by mean-centring each variable in each data table (van Velzen et al., 2008).

Index for protein and lipid signals in the 1D NMR spectra

Peaks marked L1, L2, L3, etc. in the Tables 2-3 and Supplementary Tables 4 and 8-10, are assigned to protons from fatty acyl chains of molecules usually called lipids. Such lipids are esters between long chain fatty acids and substances with one or more hydroxyl groups. The fatty acid moieties show a wide range of chain lengths and peaks from the individual chains cannot be resolved. These fatty acyl moieties can be fully saturated (i.e. R-O-CO. $(CH_2)_n$. CH_3 such palmitic acid, or mono-unsaturated with two of the CH_2 groups replaced by –CH=CH- in the cis configuration (the trans configuration is not seen naturally and is only formed by chemical processing as in “trans fats”) such as oleic acid, or with more than one double bond, i.e. polyunsaturated, such as arachidonic acid. The fatty acyl esters can be formed with many hydroxyl functions but the most common are (1) glycerol to form triglycerides (the main fat in food), but diglycerides and monoglycerides are also possible, (2) with glycerol where one or two chains are added and the third hydroxyl of glycerol is reacted with various very polar molecules such as choline phosphate to form phospholipids (those with one chain are called lysophospholipids and the chain can be on either C1 or C2 of the glycerol), and other “head groups” than choline are possible, including commonly ethanolamine and inositol, (3) carbohydrates to form gangliosides, (4) cholesterol to form cholesteryl esters, and (5) other functionality to form substances such as sphingolipids (sphingomyelin being the most common). In blood serum and plasma, these lipids (mainly triglycerides, phospholipids and cholesteryl esters) are embodied in particles called lipoproteins in various proportions. Lipoproteins can be classified according to their size and hence density into chylomicrons, low density (LDL), intermediate density (IDL) and high-density lipoproteins (HDL). Further sub-classification is possible.

In the NMR spectrum, it is not possible to resolve the individual fatty acyl groups but the different chemical environments can be resolved as described below. Moreover, some of the “lipid” peaks, particularly L1 below, show some indications of not being a single homogeneous peak and this can be deconvoluted with special software and reference to spectra from

individual lipoproteins separated by ultracentrifugation to yield information on a large range of lipoprotein sub-groups and their concentrations.

In summary, the NMR peaks can only be assigned to the chemical moieties as explained above, and not to individual molecules, nor to specific lipoproteins.

L0: Methyl group (C18, C19) from cholesterol ester: $\mathbf{CH_3}$-

L1: Methyl group: $\mathbf{CH_3}$-$(CH_2)_n$ or $\mathbf{CH_3}$-CH_2-CH_2C=

L2: Methylene group: CH_3-$\mathbf{CH_2}$-CH_2, $(\mathbf{CH_2})_n$ or CH_3-$\mathbf{CH_2}$$(CH_2)_n$

L3: Methylene group: $\mathbf{CH_2}$-CH_2-CO, $\mathbf{CH_2}$-CH_2-CH_2-CO

L4: Methylene group: $\mathbf{CH_2}$-C=C

L5: Methylene group: $\mathbf{CH_2}$-C=O

L6: Methylene group: C=C-$\mathbf{CH_2}$-C=C

L7: Methine group: **CH**=CH-CH_2-CH=CH or CH=**CH**-CH_2-CH=CH or **CH**=C-CH_2-CH_2

The NCH_2 peaks from phosphocholine containing lipids are not visible because they overlap with ethanol peaks.

Peaks from the other half of the methylene group ($\mathbf{CH_2}$OH-CH-$\mathbf{CH_2}$OH) and methine group (CH_2OH-**CH**-CH_2OH) of free glycerol are not visible because they overlap with ethanol and α-glucose peaks respectively.

Metabolite identification - Level of assignment

The level of peak overlaps in the clusters of interest and the level of confidence in the assignment of the identified metabolites were adapted from Sumner et al, (*Proposed minimum reporting standards for chemical analysis Chemical Analysis Working Group* (CAWG) Metabolomics Standards Initiative (MSI) *Metabolomics* **2007**, *3*, 211-221):

1a: Identified compound with spiking. The NMR signal not overlapped with other peaks

1.b: Identified compound with spiking but the NMR signal overlaps with other peaks, peak differentiation and quantification based upon spectral data may be compromised.

2a: Annotated compounds (without chemical reference standards, based upon physicochemical properties and/or spectral similarity with public/commercial spectral libraries). The NMR signal is not overlapped.

2.b: Annotated compounds (without chemical reference standards, based upon physicochemical properties and/or spectral similarity with public/commercial spectral libraries). The NMR signal is overlapped with other peaks. Peak differentiation and quantification based upon spectral data may be compromised

3: Putatively characterized compound classes (e.g. based upon characteristic physicochemical properties of a chemical class of compounds, or by spectral similarity to known compounds of a chemical class)

4a: Unknown compounds. The NMR signal is well defined and not overlapped

4.b: Unknown compounds. The NMR signal has low resolution, low signal/noise ratio and cannot be differentiated and quantified based upon spectral data

Online Figure S1:

Overview of study design

Online Figure S2: Confirmation of Acetaminophen (APAP)-Glucuronide assignment. A: spiking experiment with a unique concentration of APAP-glucuronide. B: spiking experiment with several concentration of APAP-glucuronide, zoom of the NMR spectra at 4.15-5.09 ppm. C: spiking experiment with several concentration of APAP-glucuronide, zoom of the NMR spectra at 3.82-3.96ppm

Online Figure S3: Confirmation of several amino acids and uridine assignments. Spiking a serum sample with a concentration of A: Aspartate, B: Phenylalanine, C:Lysine, D:5-Oxoproline, E: Glutamine, F: Uridine, G: Histidine, H: 3-Methyl-histidine, I: Methionine. For each panel, a zoom region of the ^{1}H NMR CPMG spectrum before and after spiking are displayed.

Online Figure S4: Confirmation of 1,5-Anhydrosorbitol, N,N Dimethylglycine, Ornithine, Citrulline, Phenylalanine, Tyrosine, and Histidine assignments. Spiking of serum sample with A: several concentrations of 1,5-Anhydrosorbitol, B: unique concentration of N,N Dimethylglycine and C: several concentration of Ornithine, Citrulline, Phenylalanine, Tyrosine, and Histidine. For each panel, a zoom region of the ^{1}H NMR CPMG spectrum before and after spiking are displayed.

Online Figure S5: Confirmation of Creatinine, Pyruvate, 1-Methylhistidine, Glutamate, Arginine, Uridine assignments with 2D ^{1}H-^{1}H or ^{1}H-^{13}C NMR experiments. Zoom of ^{1}H-^{13}C HSQC spectrum on A: Creatinine B: Pyruvate, C: 1-Methylhistidine. D: Zoom of ^{1}H-^{1}H COSY spectrum on a peak of Glutamate, E: Zoom of ^{1}H-^{1}H TOCSY spectrum on a peak of Arginine, F: Zoom of ^{1}H-^{1}H COSY spectrum on a peak of Uridine.

Online Figure S6:

Participant flowchart

Online Figure S7: Results from stepwise linear regression in MESA only for A)CAC and B)IMT with age, sex, cohort, ethnicity LDL and HDL, lipids and blood pressure treatment, systolic blood pressure, smoking status and diabetes forced in the model and stepwise regression of metabolites using bootrap AID with 1,000 bootstrap samples.

Online Figure S8: Ingenuity Pathway Analysis (IPA) network showing molecular relationships between metabolites which have been associated with CAC/IMT (red: inverse association, green: direct association). Metabolites that showed significant associations with CAC and/or IMT are represented by blue (inverse association with CAC/IMT) or orange (direct association with CAC/IMT) nodes, whereas the shape of the nodes represents the kind of molecule, as established in Ingenuity Knowledge Base. Solid lines indicate direct interactions, which require that two molecules make direct physical contact with each other, whereas dashed lines indicate indirect interactions.

Online Figure 1

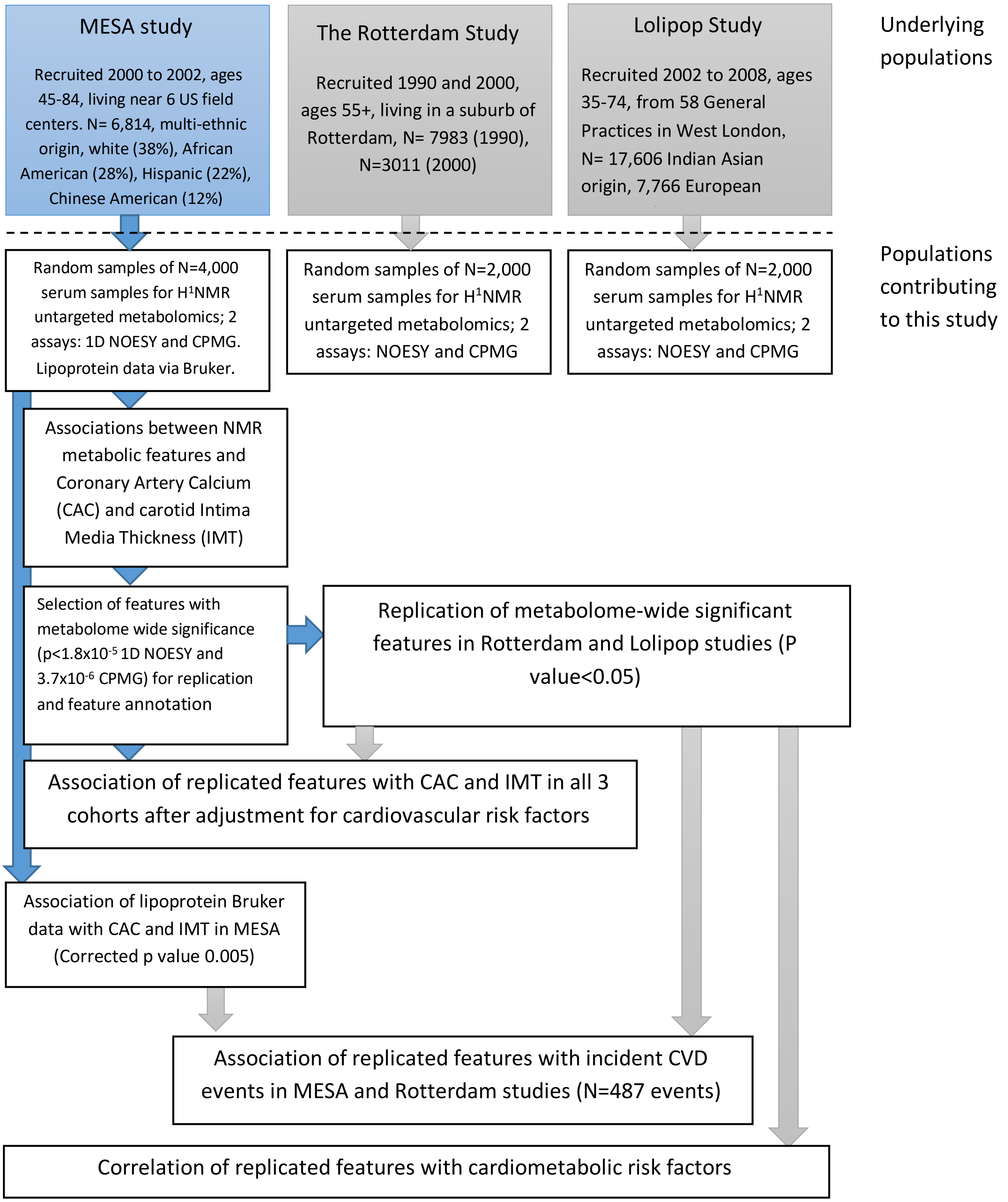

Online Figure 2

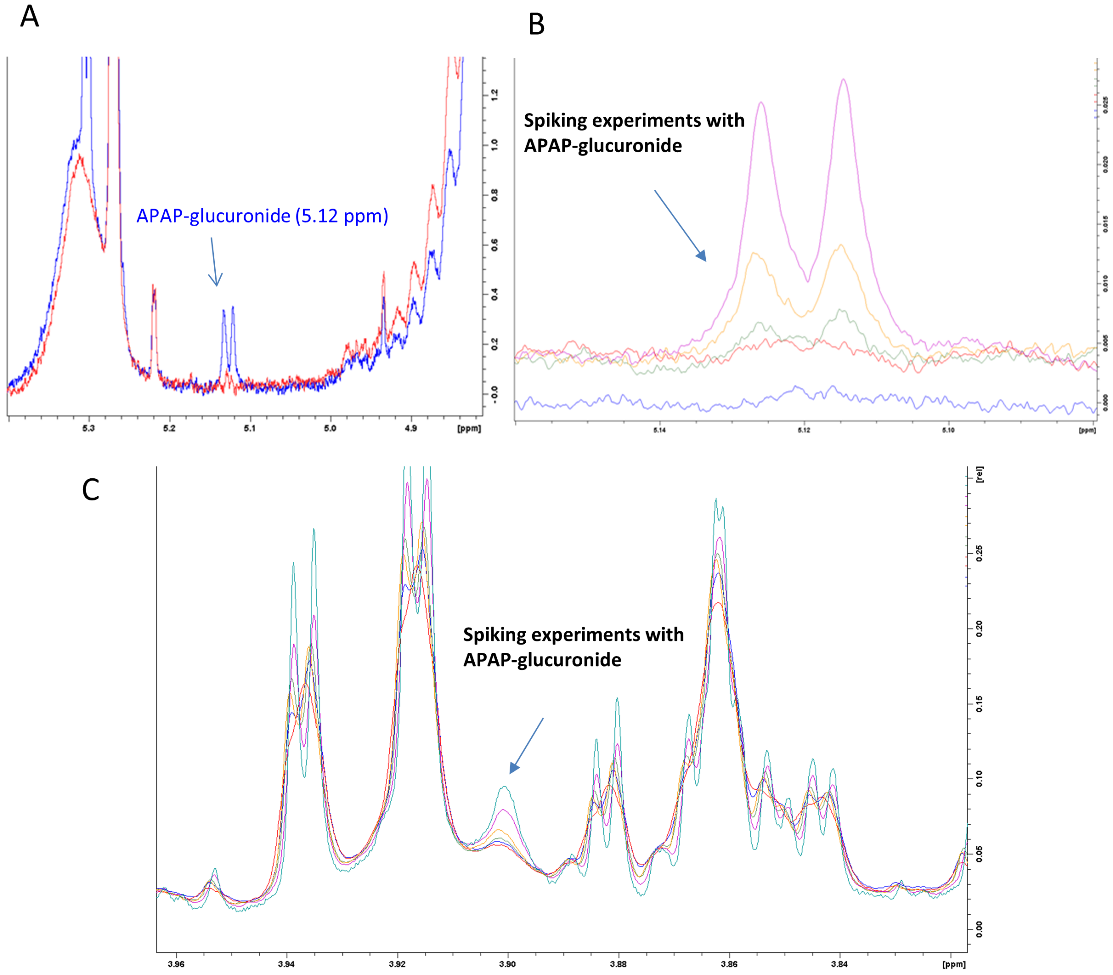

Online Figure 3

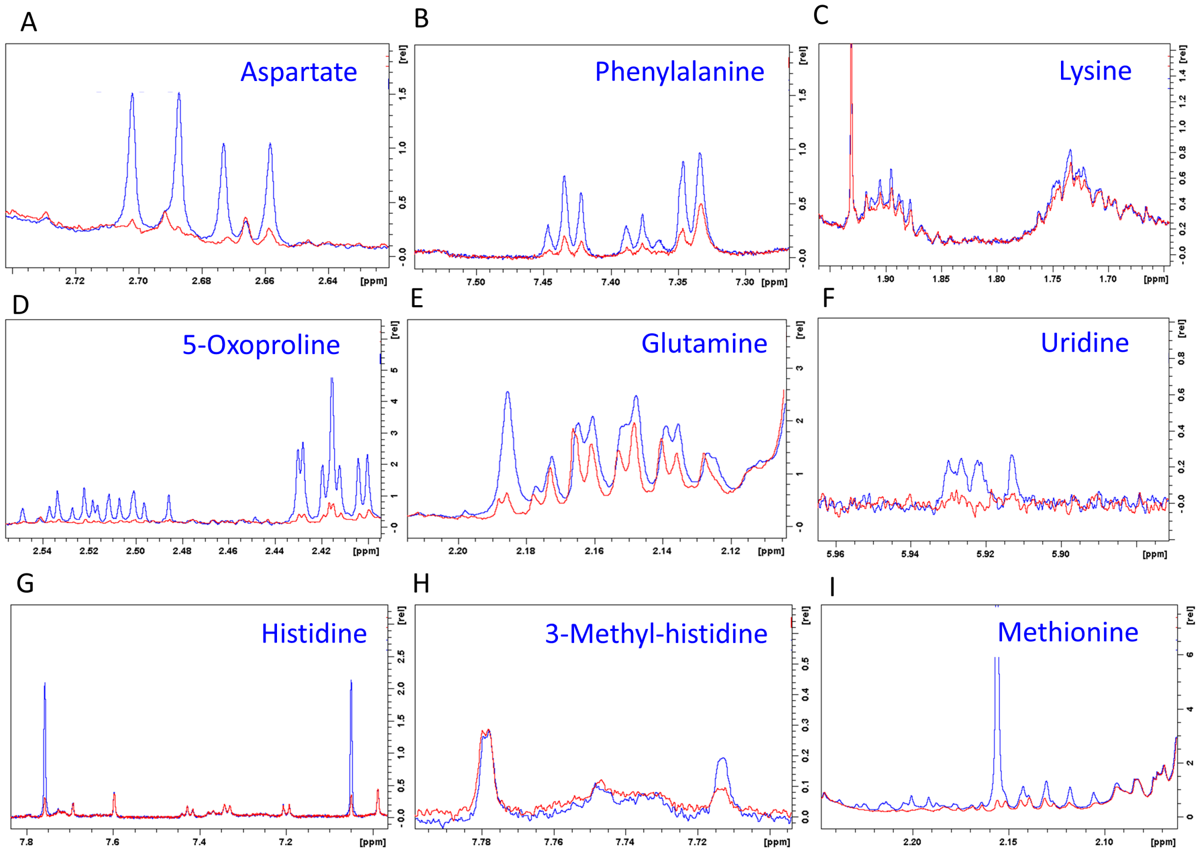

Online Figure 4

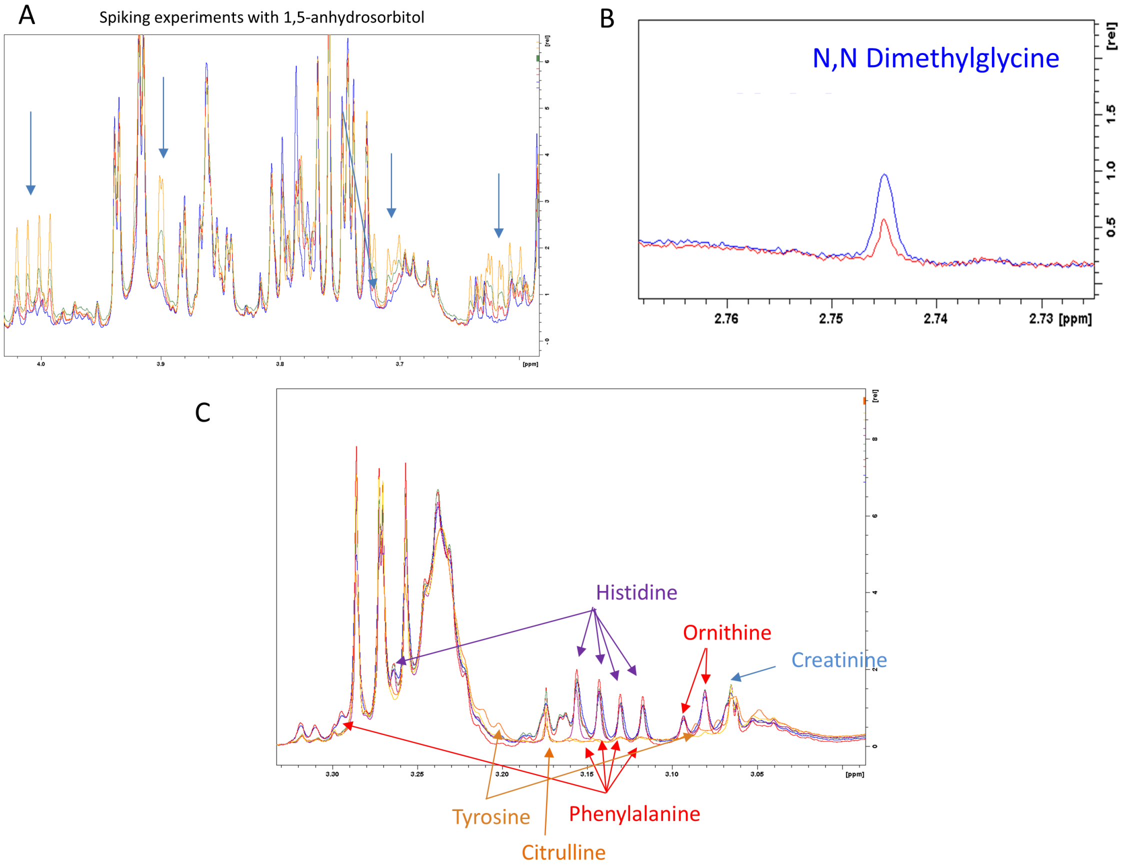

Online Figure 5

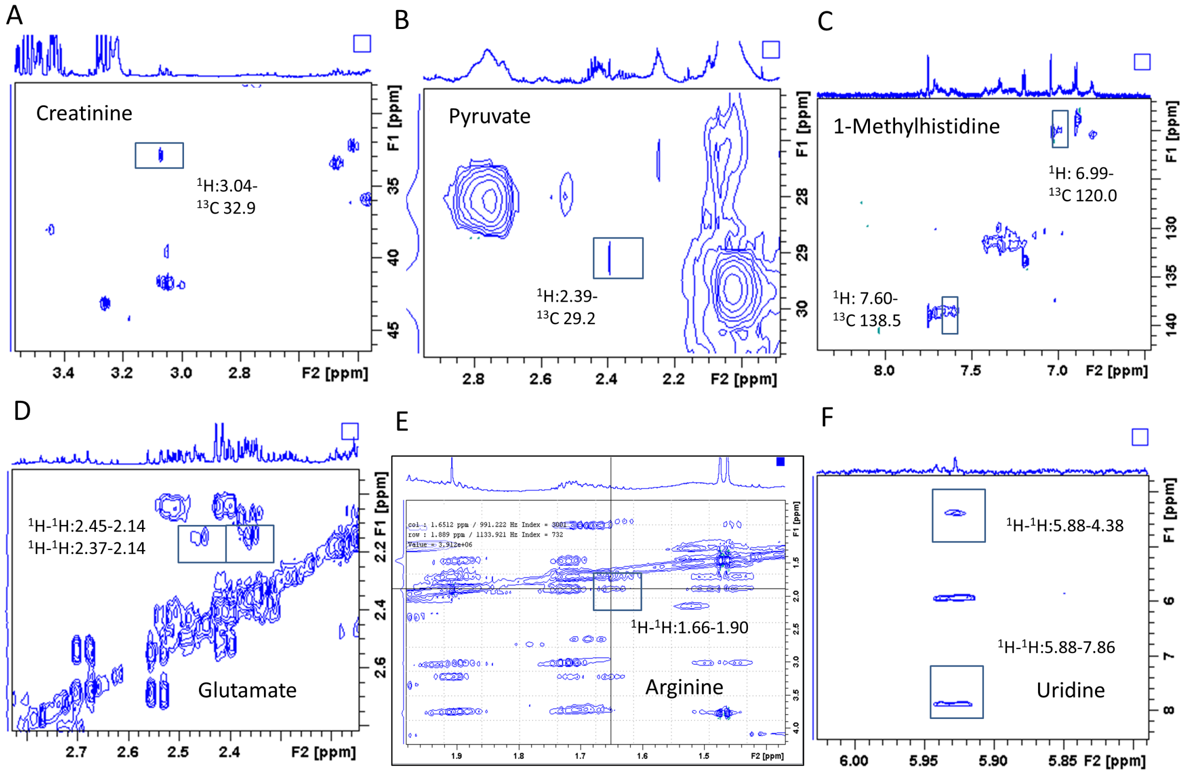

Online Figure 6

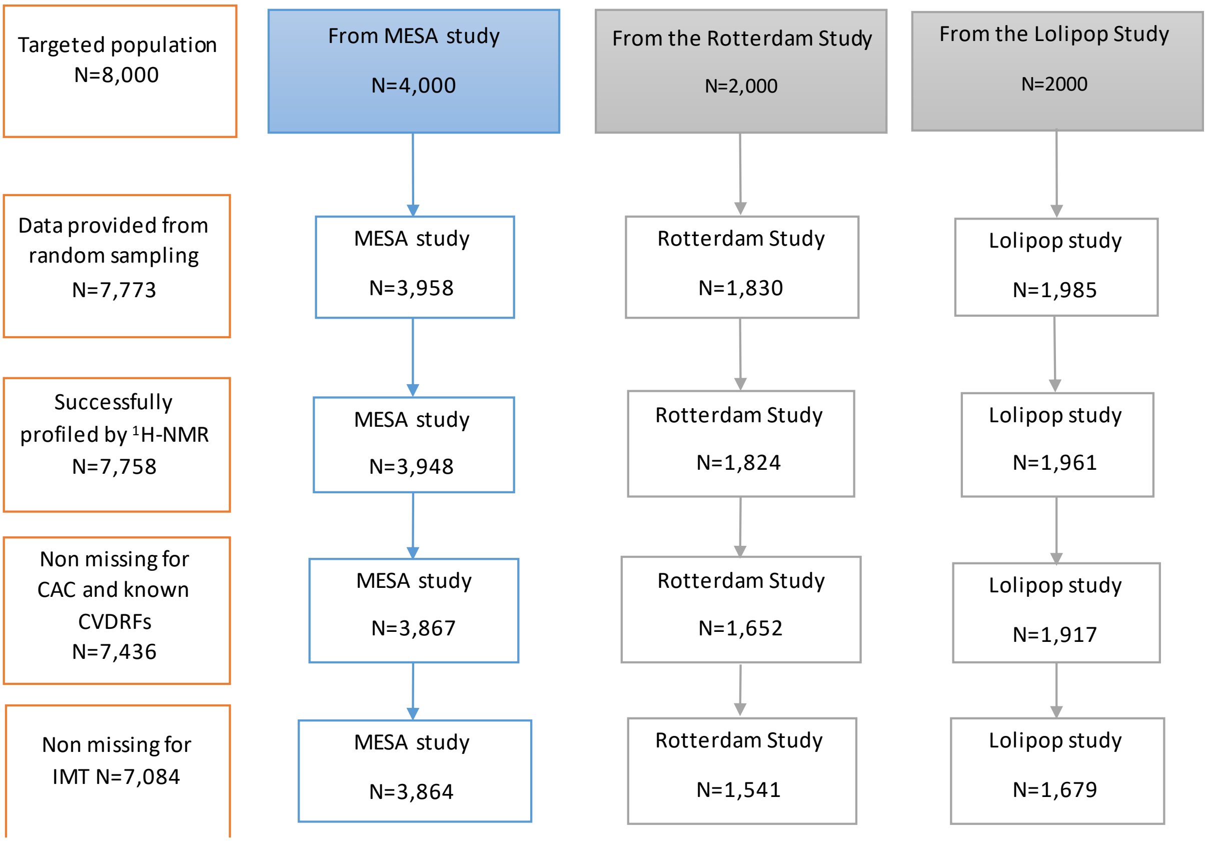

Online Figure 7

A

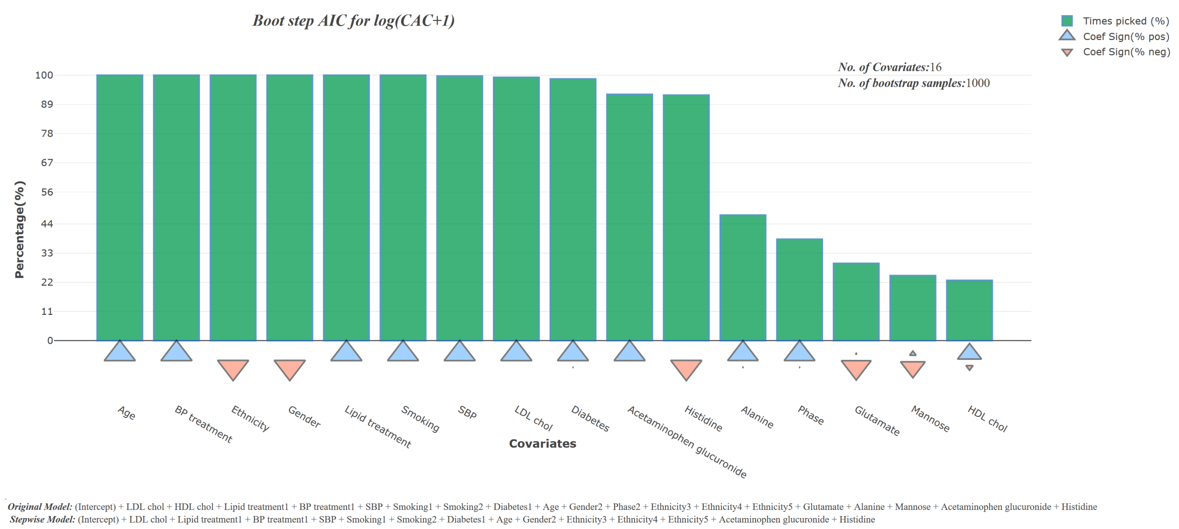

B

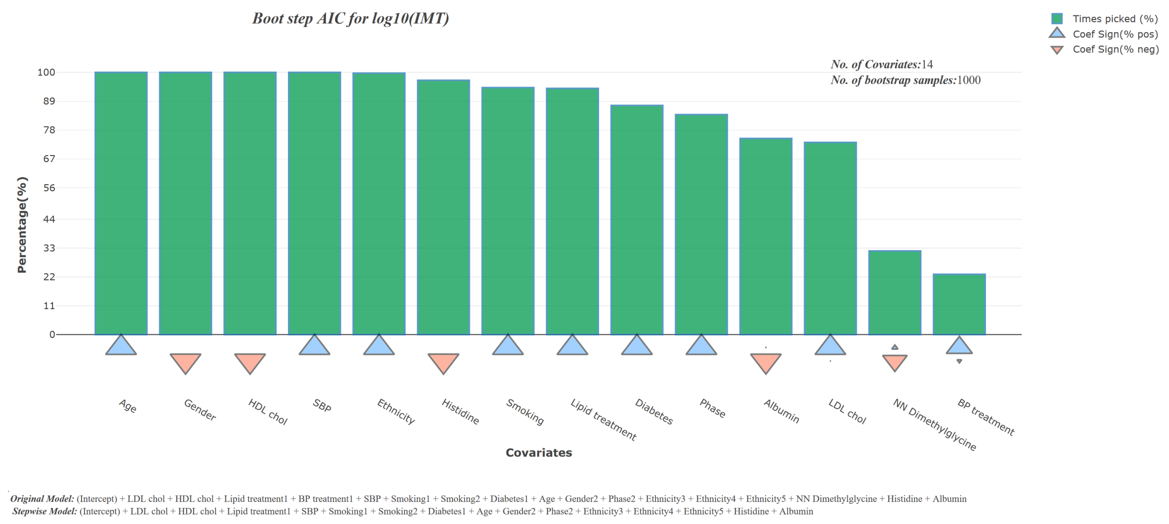

Online Figure 8

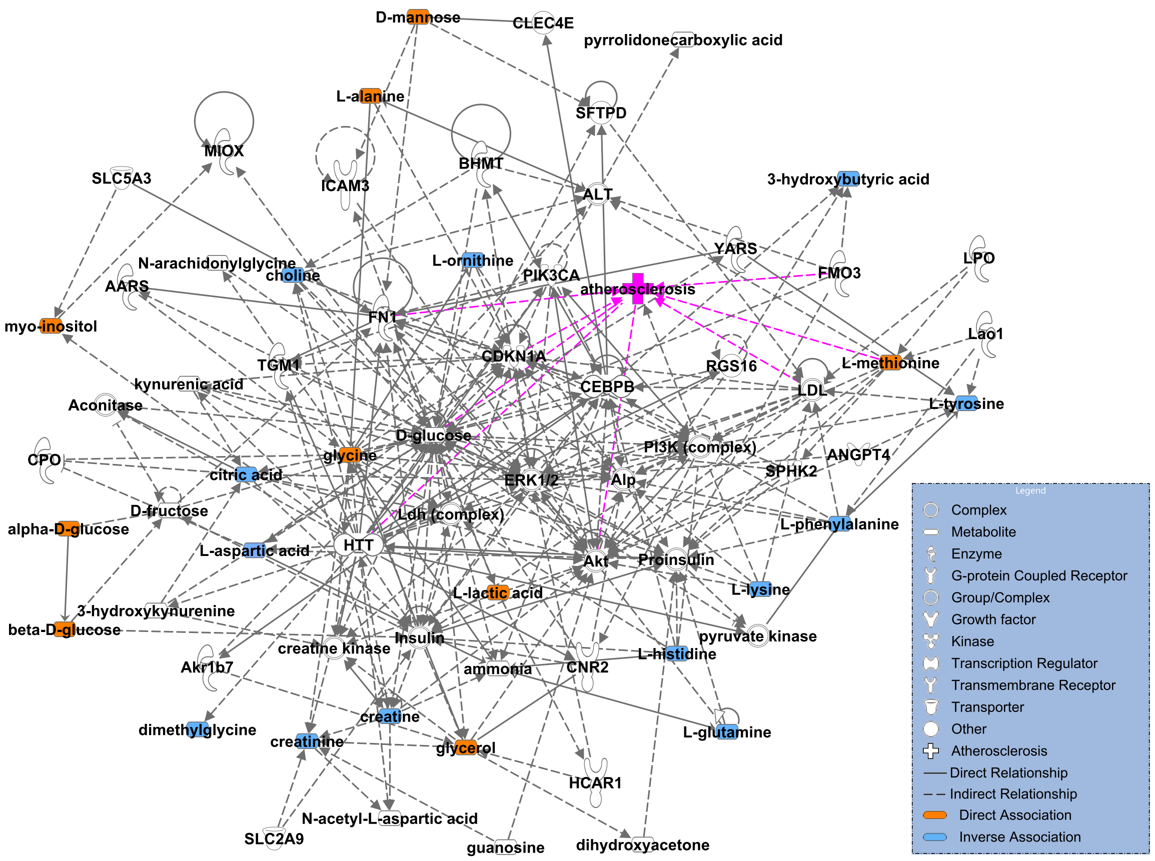

Supplement: ehz235_Supplementary_Data [file ehz235_supplementary_data.zip › ehz235-Suppl_data/Supplementary_Figures.pdf]
